# Group A Streptococcal meningitis in children: a short case series and systematic review

**DOI:** 10.1007/s10096-024-04863-2

**Published:** 2024-06-06

**Authors:** Zhen-zhen Dou, Wanrong Li, Hui-Li Hu, Xin Guo, Bing Hu, Tian-ming Chen, He-ying Chen, Ling-yun Guo, Gang Liu

**Affiliations:** 1 Department of Infectious Diseases, Key Laboratory of Major Diseases in Children, Beijing Children’s Hospital, Ministry of Education, Capital Medical University, National Center for Children’s Health, Beijing, 100045 China; 2 Research Unit of Critical infection in Children, Chinese Academy of Medical Sciences, Beijing, 2019RU016 China

**Keywords:** Bacterial meningitis, *Group A streptococcus*, Brain abscess, Otitis media, Sinusitis, Mastoiditis

## Abstract

**Background:**

Group A streptococcal(GAS) meningitis is a severe disease with a high case fatality rate. In the era of increasing GAS meningitis, our understanding about this disease is limited.

**Purpose:**

To gain a better understanding about GAS meningitis.

**Methods:**

Five new cases with GAS meningitis were reported. GAS meningitis related literatures were searched for systematic review in PUBMED and EMBASE. Case reports and case series on paediatric cases were included. Information on demographics, risk factors, symptoms, treatments, outcomes, and *emm* types of GAS was summarized.

**Results:**

Totally 263 cases were included. Among 100 individuals, 9.9% (8/81) had prior varicella, 11.1% (9/81) had anatomical factors, and 53.2% (42/79) had extracranial infections. Soft tissue infections were common among infants (10/29, 34.5%), while ear/sinus infections were more prevalent in children ≥ 3 years (21/42, 50.0%). The overall case fatality rate (CFR) was 16.2% (12/74). High risk of death was found in patients with shock or systemic complications, young children(< 3 years) and cases related to hematogenic spread. The predominate cause of death was shock(6/8). Among the 163 patients included in case series studies, ear/sinus infections ranged from 21.4 to 62.5%, while STSS/shock ranged from 12.5 to 35.7%, and the CFR ranged from 5.9 to 42.9%.

**Conclusions:**

A history of varicella, soft tissue infections, parameningeal infections and CSF leaks are important clinical clues to GAS in children with meningitis. Young children and hematogenic spread related cases need to be closely monitored for shock due to the high risk of death.

**Supplementary Information:**

The online version contains supplementary material available at 10.1007/s10096-024-04863-2.

## Introduction

Group A streptococcal (GAS) meningitis is an uncommon presentation of invasive GAS (iGAS) infection. Epidemiological studies have reported that GAS meningitis accounts for about 1% of iGAS infections across all age groups [1], and approximately 2–10% of iGAS infections in the age group < 18 years [2, 3]. Although this disease had a lower incidence than other iGAS infections, it had a higher rate of admission to paediatric intensive care unit (PICU) and case fatality rate (CFR) than other iGAS infections (except for septic shock) [2, 3]. Furthermore, this disease has been reported to have an increased incidence recently. Denmark reported a 21-fold increase in cases of adult GAS meningitis between October 13, 2022 and April 12, 2023 [4]. Netherlands reported an obviously increased GAS cases in 2022 (19 cases) and Quarter 1 2023 (10 cases), whereas the average number of GAS meningitis cases per year from 1982 to 2021 was just 5 [5]. Early diagnosis of GAS meningitis is challenging. It is hard to differentiate it from bacterial meningitis caused by other bacteria based solely on clinical symptoms. The emergence and re-emergence of various viruses causing central nervous system (CNS) infections in recent decades have highlighted the need to differentiate virus infections from GAS [6]. The patients with immunosuppression also need to consider the risk of fungal CNS infections. Nucleic acid amplification tests (NAATS) have been shown to be reliable in identifying different pathogens [6, 7], particularly when using appropriate universal polymerase chain reaction (PCR) primers [8]. The BioFire Filmarray ME Panel is the only FDA-approved multiplex PCR assay for CNS infections. It can detect 14 pathogens quickly in CSF. Its sensitivity and specificity were reported to be 90.2% (95% CI: 86.2–93.1) and 97.7% (95% CI: 94.6–99.0) respectively [9]. However, GAS is not included in this panel. Metagenomic next-generation sequencing (mNGS) is a hypothesis-free method that can detect uncommon pathogens of CNS infections (including GAS). It has been proved to be helpful in identifying causative organisms of bacterial meningoencephalitis [10, 11] and diagnosing iGAS infections [12]. Although we have had better etiological tests, our knowledge about this disease is limited. Until now, large case series studies on pediatric GAS meningitis are very limited; most of the literature about this disease were case reports. In order to gain a better understanding of this disease, we reported 5 new pediatric cases and conducted a systemic review of paediatric cases with this disease.

## Method

### Clinical information collection

We retrospectively reviewed patients (< 18 years) with GAS meningitis who were hospitalized at Beijing Children’s Hospital from January 1, 2010, to December 31, 2021. Beijing Children’s Hospital is a tertiary healthcare hospital with 970 beds. Patients who had fever, headache, signs of meningeal irritation, or altered consciousness and met any of the following criteria were defined as GAS meningitis: (1) cerebrospinal spinal fluid (CSF) culture grew GAS ; (2) blood culture grew GAS and CSF analysis showed pleocytosis [white blood cell count (WBC) > 30 × 10^6^/L] [13]; (3) GAS was identified by mNGS in CSF and CSF leukocytosis (WBC > 30 × 10^6^/L). We obtained clinical data and outcome information from the electronic medical record and follow-up records. What’s more, we had a telephone interview when this study started (June 14th, 2022). The long-term outcomes were evaluated using the Glasgow Outcome Scale–Extended Pediatric Reversion (GOS-E Peds). The GOS-E Peds scores were classified into 5 categories: 1,2 = good recovery; 3,4 = moderate disability; 5,6 = severe disability; 7 = persistent vegetative condition; 8 = death [14]. This scale had been used to evaluate the long-term outcomes of acute CNS infection in our hospital [15].

### Categories of cases

The pathogenesis of bacterial meningitis can be classified into three types. To analyze their impact on the outcomes of GAS meningitis, the cases in this study were categorized into three groups [16]. (1) Anatomical factors related cases: this group includes cases with anatomical defects, head trauma, and ventriculoperitoneal shunt (V-P shunt). The anatomical defects included CSF leaks, pilonidal sinus, and meningocele. The CNS was susceptible to bacterial invasion due to these anatomical factors. (2) Parameningeal infections related cases: this group includes cases with symptoms and/or imaging evidence of parameningeal infections but without any anatomical defects. Parameningeal infections include otitis media, sinusitis and mastoiditis. Meningitis was the result of the spread of contiguous infections. (3) Hematogenic spread related cases: this group includes cases without anatomical factors and parameningeal infections. Some cases in this category had extracranial infection focuses, such as cellulitis, arthritis, bloodstream infection, or pharyngitis. In addition, some cases had no clear local infection focus.

### Literature review and methods

We searched for relevant case reports and case series published in English in PUBMED and EMBASE. The keywords we used included “group A streptococcal meningitis”, “streptococcus” pyogenes”, “group A streptococcus”, and “meningitis”. The initial search was conducted on March 26, 2023, and an update on February 24, 2024 (showed in Online resource 1). In addition, we manually searched for references in the eligible studies. The literature inclusion criteria were: (1) case reports with detailed clinical information on pediatric GAS meningitis cases (< 18 years), (2) case series studies on pediatric GAS meningitis, (3) case series studies on invasive GAS infection, with separate descriptions of pediatric GAS meningitis, and (4) articles in English. The exclusion criteria for literature were: (1) absence of the full text, and (2) lack of detailed information about pediatric GAS meningitis cases. Two reviewers, ZZD and XG, assessed literature abstracts independently. For the articles that had different initial opinions, they reached an agreement by discussing. Data were independently extracted by ZZD, WRL. The information extracted includes the years of publication, lead author, gender, age, symptoms, risk factors, laboratory test results, diagnostic methods for identifying the clinical cause, treatments, outcomes, duration of follow-up and *emm* types of GAS. Risk factors include varicella, congenital anatomical defects in the CNS, head trauma, underlying diseases, and extracranial infections. Soft tissue infections included necrotizing fasciitis. When there were differences in the data extracted by the two workers, HHL would review the literature and make a decision. The majority of studies included were case reports, so study quality and the risk for bias were not assessed. The systematic review was not pre-registered.

### Statistical methods

Comparison of categorical variables were performed by means of χ^2^test. A P-value of < 0.05 were considered statistically significant. Statistical software used in this study was SPSS 21.0 (SPSS Inc., Chicago, IL, USA).

## Results

### Case reports

The clinical courses and head magnetic resonance imaging (MRI) of the 5 cases were summarized in Online resource 2.

#### Case 1

A previously healthy male aged 3 years 9 months was sent to the local hospital with a 3-day history of fever. Physical examination showed lethargy and meningeal irritation signs. The complete blood count (CBC) revealed a WBC count of 9.1 × 10^9^ cells/L, with 90.7% neutrophils. The C-reactive protein (CRP) level showed an increase of 272 mg/L. The CSF analysis indicated pleocytosis, with 257 × 10^6^ cells/L (73.2% neutrophils) of WBC present. The levels of CSF glucose and protein were found to be normal. He received empirical vancomycin and meropenem, however, his clinical condition worsened rapidly. On day 1, he fell into a coma, suffered frequent seizures, acute kidney injury, and shock (streptococcal shock syndrome, STSS). He was intubated and sent to the PICU. On day 3, the blood culture grew GAS, antibiotics were changed to ceftriaxone. On day 9, his intubation was discontinued due to the improved clinical conditions. He was found with muscle weakness (grade 3/5 muscle strength) in his left limbs. His head MRI revealed bilateral subdural empyema, hemorrhage on the right cerebral cortex, and substantial opacification in the bilateral sinuses. Continuous antibiotic treatments did not improve the fever, a repeated head CT on day 19 showed a subdural abscess. Therefore, ceftriaxone was switched to meropenem and linezolid. On day 40, he was sent to Beijing Children’s Hospital for the persistent fever and muscle weakness. On day 45, a repeated head MRI still revealed a subdual abscess. The patient received abscess aspiration on day 47. After that procedure, his fever and muscle strength gradually improved. On day 66, the patient was discharged from our hospital with a GOS-E Peds score of 3 (moderate disability). He was followed for 9 years. His muscle weakness recovered. The GOS-E Peds score was 1 (good recovery) at the last follow-up.

#### Case 2

An 8-year-old girl came to the local hospital with a 10-day fever and a 5-day headache, along with photophobia. Physical examination showed lethargy and meningeal irritation signs. The CBC revealed a WBC of 11.30 × 10^9^ cells/L, with 94.1% neutrophils. The CRP level was elevated to 62.28 mg/L. The CSF analysis revealed an elevated WBC of 41.00 × 10^6^ cells/L, along with normal levels of glucose and protein. The head MRI showed bright signals on diffusion-weighted imaging in the right frontal lobe. It also revealed significant opacification in the bilateral sinuses. She received vancomycin, ceftriaxone, and penicillin (the details about the treatments were unclear). On the day 3, the blood culture yielded GAS. On day 5, the patient recovered with resolution of fever and neurological symptoms. On day 14, she experienced fever again. Continuous antibiotic treatments did not improve fever. Therefore, on day 21, she was sent to Beijing Children’s Hospital for further treatment. In our hospital, the physical examination found no neurological abnormalities, arthritis, erythema, or subcutaneous nodules. A repeated CSF analysis also revealed normal results. No abscess was found in the repeated head MRI. The results of anti-streptolysin O (ASO, 1120 IU/L), erythrocyte sedimentation rate (ESR, 116 mm/H), and CRP (80.9 mg/L) revealed significantly elevated levels. The echocardiography and electrocardiography were normal. Rheumatic fever was suspected but without enough evidence. She received cefepime for 3 days and then linezolid and ceftriaxone due to fever and increased inflammation index. On day 27, her fever subsided. On day 41, she had a normal ESR and CRP. Then she was discharged from our hospital. The GOS-E Peds score was 1 (good recovery) at discharge. The patient was followed up for 5 years. No neurological sequela was found during the follow-up. The GOS-E Peds score was 1 (good recovery) at the last follow-up.

#### Case 3

A previously healthy 1 year and 6 months old male came to the hospital with a 7-day history of fever. On the day of admission, he suffered a status epilepticus, followed by coma. Eight days before admission (one day before the fever started), he burned his right hand with hot liquids. The CBC indicated a WBCs of 14.37 × 10^9^ cells/L (79.8% neutrophils), along with elevated CRP level (> 160 mg/L) and procalcitonin level (> 100 ng/ml). The CSF analysis showed a WBC of 1370 × 10^6^ cells/L and decreased glucose level (1.0 mmol/L). He received empirical meropenem. On day 3, CSF culture grew GAS. The head MRI showed extensive abnormal singles and edema in bilateral cerebral cortex. On day 10, his fever went down, but he remained in a coma with a persistent hypermyotonia. On day 23, he suffered fever again. The repeated head MRI showed left subdural effusion and ventriculomegaly. Antibiotics did not improve the fever. On day 44, he was sent to our hospital because of persistent fever and coma. During the physical examination, he was found to have a Glasgow Coma Scale score of 7/15. Persistent hypermyotonia was observed on the trunk and limbs. CBC, CRP, procalcitonin, ESR, and CSF analysis results were all normal. Echocardiography also showed normal results. Only mild pneumonia was visible in the chest X-ray. On day 50, his head CT revealed more subdural effusion compared to the previous head MRI. He received an operation to aspirate the subdural effusion on day 52. A total of 70 ml subdural effusion was drained. The results of the effusion analysis showed minimal cell counts but a significantly elevated of protein concentration (13,122 mg/L). Following the operation, there was no change in the patient’s fever, coma, or hypermyotonia. A repeated head MRI revealed extensive cortical necrosis. Considering the normal inflammation index, CSF analysis, subdural effusion analysis results, and head MRI results, the cause of the fever was suspected to be persistent hypermyotonia. On day 65, he was discharged from our hospital with a GOS-E Peds score of 7 (persistent vegetative condition). He was followed for 2 years. At the last follow-up, he was found with paralysis, cognitive impairment, and mood disorders. The GOS-E Peds score was 6 (severe disability).

#### Case 4

A previously healthy 12-year-old girl was sent to Beijing Children’s Hospital with a headache, swollen right eyelid for 3 days, fever for 2 days, and one-time convulsion. The physical examination revealed normal consciousness, signs of meningeal irritation, and a positive Babinski sign. The CBC showed a WBC of 17.6 × 10^9^ cells/L (93.2% neutrophils), along with elevated CRP level (> 160 mg/L). The CSF analysis results showed an increased number of WBC at 298 × 10^6^ cells/L, normal glucose level, and slightly elevated protein level. The head and sinus CT showed a small amount of subdural empyema and sinusitis. She was given vancomycin and ceftriaxone empirically. The fever subsided on the second day. Blood culture and CSF culture were negative. GAS (134 reads) was identified in the CSF through mNGS on the seventh day. The head MRI revealed enhancement of the leptomeninges in the right hemisphere. On day 15, the orbital abscess did not show improvement on the MRI, so we switched from vancomycin to linezolid to increase the drug concentration in the periorbital tissues. On day 18, the swollen eyelids disappeared. On day 27, the orbital abscess disappeared on the orbital CT. On day 30, the patient was discharged with a GOS-E Peds score of 1 (good recovery). She was followed for 4 years. No sequela was found and the GOS-E Peds score was 1 (good recovery) at the last follow-up.

#### Case 5

A previously healthy 11-year-old girl was sent to the local hospital due to a 6-day history of fever and headache. On the 4th day of fever, the patient had a convulsion, followed by a coma. The CBC indicated an increase in the WBC to 18.6 × 10^9^ cells/L (91.5% neutrophils), along with a CRP of 288 mg/L. The CSF analysis showed a WBC of 131 × 10^6^ cells/L, along with normal levels of glucose and protein. The head MRI revealed extensive abnormal signals in the left frontal, parietal, and occipital lobes, as well as subfalcine herniation and subdural empyema. Temporal CT revealed left mastoiditis and sinusitis, affecting the bilateral sphenoidal, ethmoid, and maxillary sinuses. She received ceftriaxone for 3 days, but there was no clinical improvement. Blood culture and CSF culture showed no growth. On day 4, she was transferred to Beijing Children’s Hospital. The physical examination showed a coma with a Glasgow Coma Scale score of 6/15 and a positive Babinski sign. No signs of meningeal irritation was detected. Empirical meropenem and vancomycin were used. Repeated CSF and blood culture also showed no growth, but the mNGS of CSF revealed GAS (41 reads). Her fever subsided on day 7. Her coma improved gradually, and she awoke on the tenth day. Hemiplegia and aphasia were found as her coma improved. On day 7, the muscle strength was graded 3/5 in the left limbs and 0/5 in the right limbs. The muscle strength improved gradually. On day 21, the patient presented with a 5/5 grade muscle strength in her left limbs and a 4/5 grade muscle strength in her right limbs. Nonetheless, her aphasia did not improve. On day 28, a repeated head MRI showed that the area of cerebral edema became smaller with improvement in subdural empyema, mastoiditis, and sinusitis, but cortical necrosis and cerebromalacia were still present. On day 30, she was discharged with a GOS-E Peds score of 5 (severe disability). She was followed up for 3 years. Three months post-hospital discharge, her muscle weakness completely recovered and she could speak a few words, but forming complete sentences was still a challenge. Seven months post-hospital discharge, she was able to speak full sentences slowly. Eighteen months post-hospital discharge, she developed symptomatic epilepsy. She was able to live independently and communicate using simple sentences. However, her academic performance was affected, leading her to enroll in a special school when her epilepsy was under control with medication. The GOS-E Peds score was 4 (moderate disability) at the last follow-up.

### Systematic review

To chart the current knowledge of GAS meningitis, we conducted a systematic review of literatures. The Preferred Reporting Items for Systematic Reviews and Meta-Analyses (PRISMA) reporting chart for this study was presented in Online resource 3. We found a total of *n* = 63 reports, including *n* = 258 patients. Of these studies, 58 reports were on individual cases [17–74] (including 95 patients); the other 5 studies were case series [75–79] (including 163 patients).

**Table 1:** Clinical information of individuals(including 95 individuals from literatures and 5 new cases in this study)

Items	Cases (%)	Number of cases^a^
Sex (male)	44 (51.2)	86
Age [median (range) ]	3 years (3 days, 17 years)	100
≤ 28 days	10 (10.0)	
>28 days, <1 year	23 (23.0)	
1–2 years	15 (15.0)	
≥ 3 years	52 (52.0)	
Symptoms		70
Fever	59 (84.2)	
Vomit	29 (41.4)	
Seizure	25 (35.7)	
Drowsiness/coma	23 (32.9)	
Headache	23 (32.9)	
Lethargy	12 (17.1)	
Earache	7 (10.0)	
Photophobia	6 (8.6)	
Pharyngodynia	6 (8.6)	
Etiological evidence		97
CSF culture	49 (50.5)	
Blood culture	7 (7.2)	
CSF and blood culture	18 (18.6)	
CSF and pus culture	3 (3.1)	
Blood and pus culture	2 (2.1)	
Blood, CSF and pus culture	2 (2.1)	
Pus culture	9 (9.2)	
Autopsy	4 (4.1)	
mNGS	2 (2.1)	
PCR	1 (1.0)	
Risk factors		81
Varicella^b^	8 (9.9)	
Anatomical factors^c^	9 (11.1)	
HIV	1 (1.2)	
Dental extraction	1 (1.2)	
Extracranial infectious lesions	42 (53.2)	79
Ear and sinus infections^d^	21 (26.6)	
Soft tissue infections^e^	11 (13.9)	
Pharyngitis	10 (12.7)	
Necrotizing enteritis	1 (1.3)	
Categories of meningitis		81
Hematogenic spread related	51 (63.0)	
< 1 year	29 (56.9)	
1–2 years	8 (15.7)	
≥ 3year	14 (27.4)	
Parameningeal infection related	21 (25.9)	
< 1 year	0	
≥ 1year and < 3 year	0	
≥ 3year	21 (100)	
Anatomic factors related	9 (11.1)	
< 1 year	0	
≥ 1year and < 3 year	2 (22.2)	
≥ 3year	7 (77.8)	
Intracranial complications		74
Subdural empyema	14 (18.9)	
Brain abscess	17 (23.0)	
Venous sinus thrombosis	2 (2.7)	
Hydrocephalus	4 (5.4)	
Cerebral infarction	3 (4.1)	
Cerebromalacia	2 (2.7)	
Systemic complication	19(26.2)	73
Respiratory failure	14 (19.2)	73
Shock^f^	12 (13.8)	87
DIC	5 (6.8)	73
Surgical treatments	29 (38.7)	75
For brain abscess	15 (51.7)	
For subdural empyema	8 (27.6)	
For other causes	6 (20.7)	
Outcome		74
Death^g^	12 (16.2)	
Hematogenic spread related cases	11 (91.7)	
Parameningeal infection related cases	1 (8.3)	
Anatomical factors related cases	0	
Survivor	62 (83.8)	
Recover without sequela	44 (71.0)	
Recover with neurological sequela	18 (29.0)	

^a^ The number of cases whose items (as showed in column 1) were reported in literatures

^b^ The duration between the onset of chickenpox and meningitis ranged from 3 to 21 days

^c^ Including 3 cases with cerebrospinal fluid leakage, 1 case with fur sinus, 4 cases with trauma, 1 case with Cochlear implant, the 4 cases with trauma include a case with wooden sick penetrated skin below the right lower eyelid and advanced to the cranium, a case with left periorbital hematoma, a case with CSF leak after head trauma, and a case with occipital skull fracture and CSF leak

^d^ Including 6 cases with otitis media, 6 cases with sinusitis, 4 cases with mastoiditis, 1 case with otitis media and mastoiditis, 1 case with otitis media, mastoiditis and sinusitis, 1case with mastoiditis and sinusitis, and 1 case with orbital cellulitis and sinusitis, therefore, the total number of cases with extracranial infection was 42

^e^ Including 1 cases with cellulitis, 1 case with paronychia, 1 case with necrotizing fasciitis, 1 case with umbilical infection, 1 case with BCG scar infection, 1 case with mandibular abscess, 1 case with hemangiomas infection and 4 cases with other soft tissue infections

^f^ Included 7 cases with streptococcal toxic shock syndrome (STSS) and 5 cases with septic shock

^g^ Including 6 cases died of shock or STSS, 1 case died of cerebral herniation, 1 case died of severe brain damage, 4 cases didn’t describe the cause of death. 7 of 12 deaths younger than 1 years

CSF: cerebrospinal fluid; STSS: Streptococcal toxic shock syndrome

#### Demographics and symptoms of individual cases

Findings from reports on individual cases and 5 new patients in this study (100 cases in total) were summarized in Table 1.The detailed information on individuals was provided in Online resource 4. Among these 100 patients, the infants accounted for 33.0% (33/100). Sixty-eight individual cases described symptoms. Fever, vomit, and seizures were the most common clinical manifestations (84.2%, 41.4%, and 35.7%, respectively). In 10.0% (7/70) of individual cases, there was a preceding earache.

#### Risk factors and categories of meningitis in individual cases

Only 19 individual cases (19%) had no description about risk factors. Among the other 81 individual cases, varicella (8/81, 9.9%) and anatomical factors (9/81, 11.1%) were the most common preceding risk factors. Most cases of GAS meningitis were observed between days 3 and 5 of varicella lesions (*n* = 5), while one case occurred on day 7 and two cases occurred after 3 weeks of varicella. The anatomical factors consist of 3 instances of CSF leak, 1 instance of pilonidal sinus, and 4 instances of head trauma. The 4 cases of head trauma included a case where a wooden sick pierced the skin below the right lower eyelid and reached to the cranium, a case of a left periorbital hematoma, a case of CSF leak after head trauma, and a case of occipital skull fracture with CSF leak. Extracranial infectious focuses were observed in 53.2% (42/79) of individual cases. As displayed in Table 2, among infants, the most prevalent type of extracranial infection focus was soft tissue infection (10/29, 34.5%), while among children aged 3 years or older, the most common extracranial infections were ear, sinus and mastoid infection (21/42, 50.0%).

According to the information on risk factors, 81 cases were classified into three categories based on the causes of meningitis. As shown in Table 1, the majority (51/81, 63.0%) were associated with hematogenic spread, followed by 25.9% (21/81) with parameningeal infection, and 11.1% (9 out of 81) with anatomical factors. Among the 51 cases related to hematogenic spread, infants accounted for 56.9% (29/51), followed by cases aged 3 years or older (27.4%, 14/51), and cases aged 1–2 years (15.7%, 8/51). All 21 cases related to parameningeal infection were aged 3 years or older.

**Table 2:** Risk factors, extracranial infection and outcomes in different age groups of individuals (including 95 individuals from literatures and 5 new cases in this study)

Age groups (CFR^a^)	Risk factors	Numbers	Outcomes
< 1year		33	
(7/25, 28.0%)	Soft tissues infections^b^	10 (10/29, 34.5%)	Recover (7), death (1), ND (1), sequela (1)
	Chickenpox	2 (2/29, 6.9%)	Recover (1), sequela (1)
	Necrotizing enterocolitis	1 (1/29, 3.5%)	Death (1)
	No local infection or other risk factors	16 (16/29, 55.2%)	Recover (4), sequela (4), death (5),ND (3)
	ND	4	ND (4)
1-2years		15	
(3/10, 30.0%)	Pharyngitis	2 (2/10, 20.0%)	Recover (2)
	Anatomic factors^c^	2 (2/10, 20.0%)	Recover (2)
	Varicella	3 (3/10, 30.0%)	Recover (1), death (2)
	Scalded on right hand	1 (1/10, 10.0%)	Sequela (1)
	No local infection or other risk factors	2 (2/10, 20.0%)	Recover (1), death (1)
	ND	5	ND (6)
≥ 3years		52	
(2/39, 5.6%)	Parameningeal infection	21 (21/42, 50.0%)	Recover (12), sequela (7), death (1),ND (1)
	Anatomic factors	7 (7/42, 16.7%)	Recover (4), sequela (1),ND (2)
	Pharyngitis	7 (7/42, 16.7%)	Recover (5), sequela (1),ND (1)
	Varicella	2 (2/42, 4.7%)	Recover (2)
	Dental extraction	1 (1/42, 2.4%)	Sequela (1)
	Cellulitis	1 (1/42, 2.4%)	Recover (1)
	No local infection or other risk factors	3 (3/42, 7.1%)	Recover (2), death (1)
	ND	10	Sequela (1), ND (9)

^a^ CRF = No. of death/No of case with outcome information

^b^ including BCG scar infection (1), hemangiomas infection (1), Mandibular abscess (1), necrotizing fasciitis (1), soft tissues infection in foot (1), soft tissues infection in lower limbs (1), soft tissues infection in wrists and ankle (1), umbilical infection (1), paronychia (1) and right forearm infection (1)

^c^including 1 case with cerebrospinal fluid leakage and 1 case with pilonidal sinus

ND: no description

#### Evidence of GAS and treatments for individual cases

Evidence of GAS was observed in 97 individual cases. Among them, 74.2% (72/97) had GAS present in their CSF, 29.9% (29/97) had GAS present in their blood, 25.8% (25/97) had GAS cultured from pus in various local infectious sites, and 5.1% (5/97) of had GAS cultured from autopsy specimens. The antibiotic treatments differed among various studies, with 42 individuals using penicillin, 49 using ceftriaxone or cefotaxime, and 18 using vancomycin or linezolid. Twenty-nine out of seventy-six individuals (38.2%) received surgical treatment, and 51.7% of the surgeries were performed to address brain abscesses. The detailed information about treatments is shown in Online resource 4.

#### Complications and outcomes of individual cases

Among patients with complication information, 26.2%(19/73) had systemic complications, 23% (17/74) had brain abscesses, and 18.9% (14/74) had subdural empyema. Eighty-seven cases had information on shock or STSS, with 13.7% (12/87) of them complicated by shock or STSS.

Seventy-four individual cases were reported outcomes in literatures, including 12 deaths. The overall CRF was 16.2% (12/74). Patients with shock(6/10 vs. 2/56, *P* < 0.001), systemic complications(5/14 vs. 3/51, *P* = 0.11) and children aged < 3 years (10/35 vs. 2/39, *P* = 0.016) had a higher a risk of death. Hematogenic spread related cases accounted for 11 out of the 12 deaths. Cases in this category also tended to have a higher risk of death (11/46 vs. 1/27, *P* = 0.055). Detailed statistical information was shown in Online resource 6.

Among the 12 deaths, the specific causes of death for 4 individuals were not stated. Among the other 8 cases, it was revealed that 6 died from shock or STSS, 1 from cerebral herniation, and 1 from severe brain damage; 8 deaths occurred within 24 h of admission, 2 deaths occurred in home. Among the 62 survivors (Table 1), 18 (18/62, 29.0%) of them recovered with neurological sequela (Online resource 5), including: sensorineural deafness (*n* = 4), symptomatic epilepsy (*n* = 4), developmental delay (*n* = 4), epilepsy and developmental delay (*n* = 1), limb movement disorders (*n* = 5), visual impairment (*n* = 4), learning difficulty (*n* = 1), cranial nerve palsy (*n* = 1), partial nominal aphasia (*n* = 1), spatial orientation and deficit in short-term memory (*n* = 1), mood disorders (*n* = 1), headache and possible attention disorder (*n* = 1), 10 survivors had multiple neurological sequela. One case with hypomyotonia completely recovered during the follow-up period; 34 survivors described follow-up duration in literatures, the duration of follow-up ranged from 2 weeks to 9 years.

#### Clinical information from case series studies

Clinical information of 5 included case series studies was summarized in Table 3; the number of cases varied among the studies, ranging from 8 to 83. The incidence of infants ranged from 22 to 56%. The most common extracranial infections were ear, sinus and mastoid infection (ranging from 21.4 to 62.5%). STSS or shock were common (ranging from 12.5 to 35.7%), and the CFR was high in the most of studies (ranging from 5.9 to 42.9%). The proportion of cases related to hematogenic spread ranged from 37.3 to 78.6%, the proportion of cases related to parameningeal infections ranged from 21.4 to 41.2%, and the proportion of cases related to anatomical factors ranged from 0 to 21.7%. The information on risk factors for GAS meningitis in different age groups could be accessed in two case series studies [75, 76], where all cases related to parameningeal infections were aged 3 years or older. Similar to individual cases, hematogenic spread related cases also be found had a higher CFR in cases series studies. In Link-Gelles R’s study, hematogenic spread related cases had a CFR of 17%, which was higher than ear, nose, throat (ENT) -related cases (9%) and ventriculoperitoneal shunt (V-P shunt) infection (0%).

**Table 3:** Clinical information of cases from case series

Publish time /Country	Author	NO. of cases	age median (range)	sex	Extracranial foci or other risk factors	Categories of meningitis	Complication	Death (CRF)	emm types
2009/ Brazil	Santos MS	16	2.3Y (19d-17Y), including 9 (56.3%) cases younger than 1 year, the other 7 cases older than 3 years	F: 7 M: 9	Otitis media: 4 (25.0%) Pharyngitis: 3 (18.6%) Head trauma: 1 (6.3%) V-P shunt: 1 (6.3%) meningomyelocele: 1 (6.3%)	Primary infection: 10 (62.5%) Parameningeal infection related: 4 (25.0%) Anatomic factors related: 2 (12.5%)	ND	4 (25.0%)	All 16 had serotyping: : *emm*11 (*n* = 1), *emm*25 (*n* = 3), *emm*39.1 (*n* = 1), *emm*55 (*n* = 1), *emm*73 (*n* = 2), *emm*81 (*n* = 1), *emm*85 (*n* = 1), emm86.1 (*n* = 1), *emm*92 (*n* = 1), *emm*95 (*n* = 1),*emm*112 (*n* = 1), *emm*118 (*n* = 1),*emm*123 (*n* = 1)
2013/ Brazil	de Almeida	14	3.5Y (21d-11Y), including 5 (35.7%) cases younger than 1 year, 7 cases older than 3 years	F: 8 M: 6	Otitis media: 3 (21.4%) Pneumonia: 1 (7.1%) Varicella: 1 (7.1%)	Primary infection: 11 (78.6%) Parameningeal infection related: 3 (21.4%) Anatomic factors related: 0	Seizure: 3 STSS: 5	6 (42.9%)	All 14 had serotyping: : *emm*1 (*n* = 2), *emm*6 (*n* = 2), *emm*12 (*n* = 3), *emm*66 (*n* = 1), *emm*75 (*n* = 1), *emm*83 (*n* = 2), *emm*89 (*n* = 1), *emm*92 (*n* = 1) and *emm*112 (*n* = 1)
2013/ France	Levy C	34	5.6Y (1.3 m-14.2Y)	F: 12 M: 2	Otitis media: 14 (41.2%) Pharyngitis: 6 (17.6%)	Primary infection: 20 (58.8%) Parameningeal infection related: 14 (41.2%) Anatomic factors related: 0	ND	2 (5.9%)	12 of 34 stains had available serotyping, including: *emm*1 (*n* = 4), *emm*6 (*n* = 3), *emm*3 (*n* = 1), *emm*12 (*n* = 1), *emm*28 (*n* = 1), *emm*44 (*n* = 1) and *emm*102 (*n* = 1)
2016/ Australia	Tuerlinckx D	8	5.8Y (3 m-9.75Y)	F: 3 M: 5	Otitis media: 5 (62.5%) Mastoiditis: 4 (50.0%) Pharyngitis: 1 (12.5%) Sinusitis: 1 (12.5%) Necrotizing fasciitis: 1 (12.5%)	Can’t be classified^a^	STSS: 1	0	All 8 had serotyping: : *emm*1 (*n* = 4), *emm*6 (*n* = 2), *emm3* (*n* = 1), *emm5* (*n* = 1)
2020/USA	Link-Gelles R	83	22.0% of cases younger than 1 year, the range of age was 4d-15Y	ND	ENT related: 34 (41.0%) V-P shunt: 14 (16.9%) septic arthritis: 3 (36.1%) Trauma: 7 (8.4%) (including 3 head trauma) cochlear implant: 1 (1.2%)	Primary infection: 31 (37.3%) Parameningeal infection related: 34 (41.0%) Anatomic factors related: 18 (21.7%) (14 VP shunt, 3 head trauma and 1 cochlear implant)	STSS: 7 Septic shock: 2 Intracranial abscess: 4 epidural empyema: 2 subdural empyema: 5	14 (15.4%)	65 of 91 stains had available serotyping: : *emm*1 (n = 20), *emm2 (n = 1), emm3 (n = 2),emm4 (n = 3), emm5* (n = 2), *emm*6 (n = 4), *emm*9 (n = 1), *emm*11 (n = 2), *emm*12 (n = 9), *emm*18 (n = 1), *emm*28 (n = 4), *emm*44 (n = 1), *emm*49 (n = 1), *emm*59 (n = 2), *emm*75 (n = 2), *emm*77 (n = 1), *emm*86 (n = 1), *emm*87 (n = 1), *emm*89 (n = 3), *emm*118 (n = 3) and *emm*122 (n = 1)

^a^The total number of extracranial infection foci were larger than the number of cases, maybe some cases had multiple extracranial infection foci, therefore, cases can’t be classified according to information in literature, maybe the most of them were parameningeal infection related cases. ND: no description

#### Distribution of emm types

**Fig. 1:**
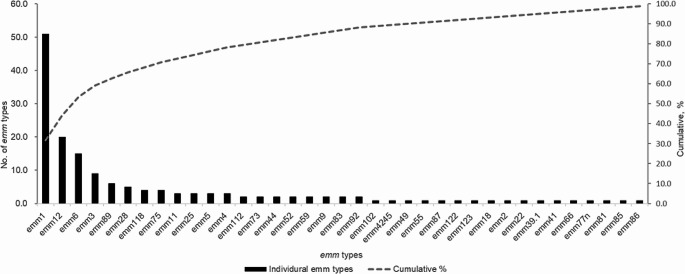
Distribution of *Group A streptococcus* isolates reported in literatures

Among all reports on individual cases and case series studies, a total of 161 isolates were identified with *emm* types, the *emm* types distribution was shown in Fig. 1. The most frequent types were *emm*1 (51/161, 31.7%), *emm*12 (20/161, 12.4%), *emm*6 (15/161, 9.3%), and *emm*3 (9/161, 5.6%), collectively accounting for 59.0% (95/161) of isolates.

## Discussion

In this study, around 2/3 of individual cases and approximately 50–80% of cases reported in case series studies older than 1 year. However, in a multi-center retrospective study from China, only 1/4 of meningitis cases (mainly caused by *Streptococcus pneumoniae, group B streptococcus*, and *Escherichia coli*) were in this group [79]. The CFR of individual cases in this study was nearly 17% (Table 1), which was similar to the findings of the largest case series study conducted in the USA [16] (15.4%). Additionally, two studies on the epidemiology of iGAS infection in the USA even revealed higher CFRs of 24% and 27% for GAS meningitis cases < 10 years [80, 81]. However, another study on bacterial meningitis caused by other pathogens in the USA reported an obviously lower CFR of 2.8% [82]. Among the survivors of individual cases, more than one-quarter were found to have neurological sequelae. This risk was higher compared to patients with *Haemophilus influenzae* type b meningitis (14.5%) and Meningococcal meningitis (9.5%), but lower compared to those with *Pneumococcal* meningitis (34.7%) [83]. Meningitis caused by GAS tends to occur more frequently in older children and has a worse outcome compared to meningitis caused by other bacteria.

Previous researches have shown a significant link between varicella zoster virus (VZV) infection and the risk of developing iGAS infection [84], usually leading to skin and soft tissue infections [40, 85]. In this study, almost 10% of individual cases were found with preceding varicella, nearly 40% of the individual infants had a concurrent soft tissue infection (Table 2), highlighting the skin as an important entry point for GAS, particularly in infants. To lower the risk of GAS meningitis in young children, it is essential to prioritize the VZV vaccine and maintain proper skin care.

Nearly 20% of individual cases were reported to have pharyngitis before the onset of GAS meningitis. Most of them were aged ≥ 3 years. Previous study has also shown that GAS is commonly found in the throats of adults with iGAS infections [86]. Additionally, a 15-year time-series analysis revealed an increase in iGAS cases in children during respiratory virus outbreaks [87]. It is likely that respiratory plays a significant role in invasion of GAS, leading to the spread of GAS through the bloodstream. It is essential for physicians to be watchful for GAS meningitis, especially during respiratory virus pandemics.

We found that all infants and over 80% of cases in the 1–2 years age group were hematogenic spread related cases. In older children, parameningeal infections became more prevalent. Among individual cases aged ≥ 3 years, half were related to parameningeal infection. The pathogenesis of meningitis differs between young and older patients. Most young patients suffered from GAS meningitis as a result of GAS hematogenous seeding. In older children, the development of GAS meningitis was similar to that in adults [88], otitis and sinusitis played a more important role in developing GAS meningitis.

Similar to meningitis caused by other bacteria, symptoms like fever, vomiting, seizures, headache, lethargy, and coma were common among cases of GAS meningitis in this study. Therefore, it is hard to distinguish GAS meningitis from meningitis caused by other bacteria based on meningitis symptoms. However, it is worth noting that over 3/4 of individual cases in this study (Table 1) were complicated with extracranial infections or had preceding high risk factors. These clinical clues may prompt us to suspect GAS meningitis. What’s more, another five cases were found to have CSF leaks (3 congenital defects, 2 post-trauma). GAS can colonize in the pharynx, and it is a common cause of ear infections and sinus infections. It’s easy to understand that GAS can cause meningitis when patients have ear or sinus infections, or a CSF leak. In fact, *Streptococcus pneumoniae* was reported as the most common pathogen of otogenic meningitis, sinusitis related meningitis, and CSF leaks related meningitis in a previous study [89]. During the era of pneumococcal conjugate vaccines (PCV), there has been a decline in the cases of pneumococcal acute otitis media [90]. The cases of otogenic meningitis caused by *Streptococcus pneumoniae* have decreased significantly among children under 2 years old [91]. In contrast, there has been an increase in GAS meningitis incidence [4, 5]. Therefore, patients who have been vaccinated with PCV should be cautious of GAS if they develop meningitis from CSF leaks or infections in the ear or sinuses.

As shown in Table 1, most individual cases were confirmed to have GAS through culturing, and two more cases confirmed by mNGS (case 4 and case 5). Although mNGS has revealed a higher sensitivity than culture [92], this detection method needs relatively long turnaround time (1–2 days), costs more money, and has lower sensitivity than NAATS. What’s more, the results of mNGS must be interpreted in the clinical context. The two cases we reported in this study presented with parameningeal infections and typical meningitis symptoms such as headache, lethargy, seizures, and meningeal irritation. Their CSF analysis showed pleocytosis, and their head MRI revealed enhanced leptomeninges. Their etiological examinations (blood culture, CSF culture, and rapid antigen detection test for *Streptococcus pneumonia*) were negative. No other organisms were detected except for GAS with mNGS. Therefore, we identified GAS as the causative pathogen in the two cases.

Positive GAS DNA analysis of CSF also helps us confirm more cases. A study from Denmark, which involved adult cases with positive GAS DNA analysis of CSF [4], found a higher incidence than that reported from the Netherlands (only cases with positive CSF culture were included) [5]. Given the increasing cases of iGAS infections, it could be beneficial to consider incorporating GAS-specific PCR as a routine detection for patients with meningitis, especially those displaying clinical clues of GAS infection (such as soft tissue infections, parameningeal infections, varicella, and CSF leaks).

Rapid antigen detection tests (RADTs) for GAS are a fast, inexpensive, and convenient method for diagnosing acute pharyngitis. Several studies have confirmed that it also has high sensitivity and specificity when used in specimens from deep tissue [93], pleural fluid [94, 95], and ear fluid [96]. As shown in Table 1, a significant portion of GAS meningitis occurs concurrently with soft tissue infection or otitis media. RADTs could be used to detect GAS with specimens from these infectious focuses. Considering its high specificity and convenience of detection (it can be performed in < 1 h), this method shows promise for diagnosing GAS meningitis, especially in resource-limited environments.

Among individual cases, young children(< 3 years)were found a higher risk of death(*P* = 0.016). A similar trend was also observed among cases related to hematogenic spread(*P* = 0.055). Similar findings were also reported in the case series study by Link-Gelles R [16]. In 8 cases with known causes of death, shock or STSS accounted for 6 of the deaths, highlighting the significant role of shock in causing death. Deaths typically occurred in the first few days, 3/4 of deaths occurred within 24 h of admission. The rapid deterioration of GAS meningitis makes the diagnosis and treatment challenging. Early clinical suspicion of GAS meningitis, appropriate antibiotics, aggressive hemodynamic monitoring and timely anti-shock treatments are crucial to improve the patient’s chances of survival, especially for children aged < 3 years and hematogenic spread related cases.

Special distribution of *emm* types was not found in the cases of GAS meningitis. The most common *emm* types were *emm*1, *emm*12, *emm*6, and *emm*3, which were similar to other iGAS infections reported in previous studies [80, 81].

The limitation of this study is that publication bias may have affected the data from reports on individual cases. Due to the rarity of GAS meningitis, most of the clinical data available comes from case reports and small case series. However, we found that the data from individual cases regarding age distribution, risk factors and CFR were generally in line with the findings of large epidemiological studies on iGAS infections. To gain a deeper insight into this disease, more prospective cohort studies are necessary.

To conclude, a history of varicella, soft tissue infections, parameningeal infections and CSF leaks were important clinical clues to suspect GAS in children with meningitis, especially for those who have been vaccinated with PCV. Children < 3 years and hematogenic spread related cases need to be closely monitored for shock due to the high risk of death.

### Electronic supplementary material

Below is the link to the electronic supplementary material.

Supplementary Material 1

Supplementary Material 2

Supplementary Material 3

Supplementary Material 4

Supplementary Material 5

Supplementary Material 6

## Data Availability

The datasets analysed during the current study are available from the corresponding author on reasonable request.

## Abbreviations

ASO: Anti-streptolysin O
CBC: Complete blood count
CFR: Case fatality rate
CNS: Central nervous system
CRP: C-reactive protein
CSF: Cerebrospinal fluid
ENT: Ear, nose, throat
ESR: Erythrocyte sedimentation rate
GAS: Group A streptococcus
GOS-E Peds: Glasgow Outcome Scale–Extended Paediatric Reversion
iGAS: Invasive group A streptococcus
mNGS: Metagenomic next-generation sequencing
MRI: Magnetic resonance imaging
NAATS: Nucleic acid amplification tests
PCR: Polymerase chain reaction
PCV: Pneumococcal conjugate vaccines
PICU: Paediatric intensive care unit
PRISMA: Preferred Reporting Items for Systematic Reviews and Meta-Analyses
RADTs: Rapid antigen detection tests
STSS: Streptococcal shock syndrome
V-P shunt: Ventriculoperitoneal shunt
VZV: Varicella zoster virus
WBC: White blood cell
