# Supplementary material for: Group A Streptococcal meningitis in children: a short case series and systematic review
Source: Eur J Clin Microbiol Infect Dis. 2024 Jun 6;43(8):1517–31. doi: 10.1007/s10096-024-04863-2 (PMC11271352; doi:10.1007/s10096-024-04863-2)

The European Journal of Clinical Microbiology & Infectious Diseases

Group A Streptococcal Meningitis in Children: A Short Case Series and Systematic Review

Zhen-zhen Dou MD, Wanrong Li MMed, Hui-Li Hu, MBBS, Xin Guo, MMed, Bing Hu, MMed, Tian-ming Chen, MMed, He-ying Chen, MBBS, Ling-yun Guo, MD, Gang Liu, MD

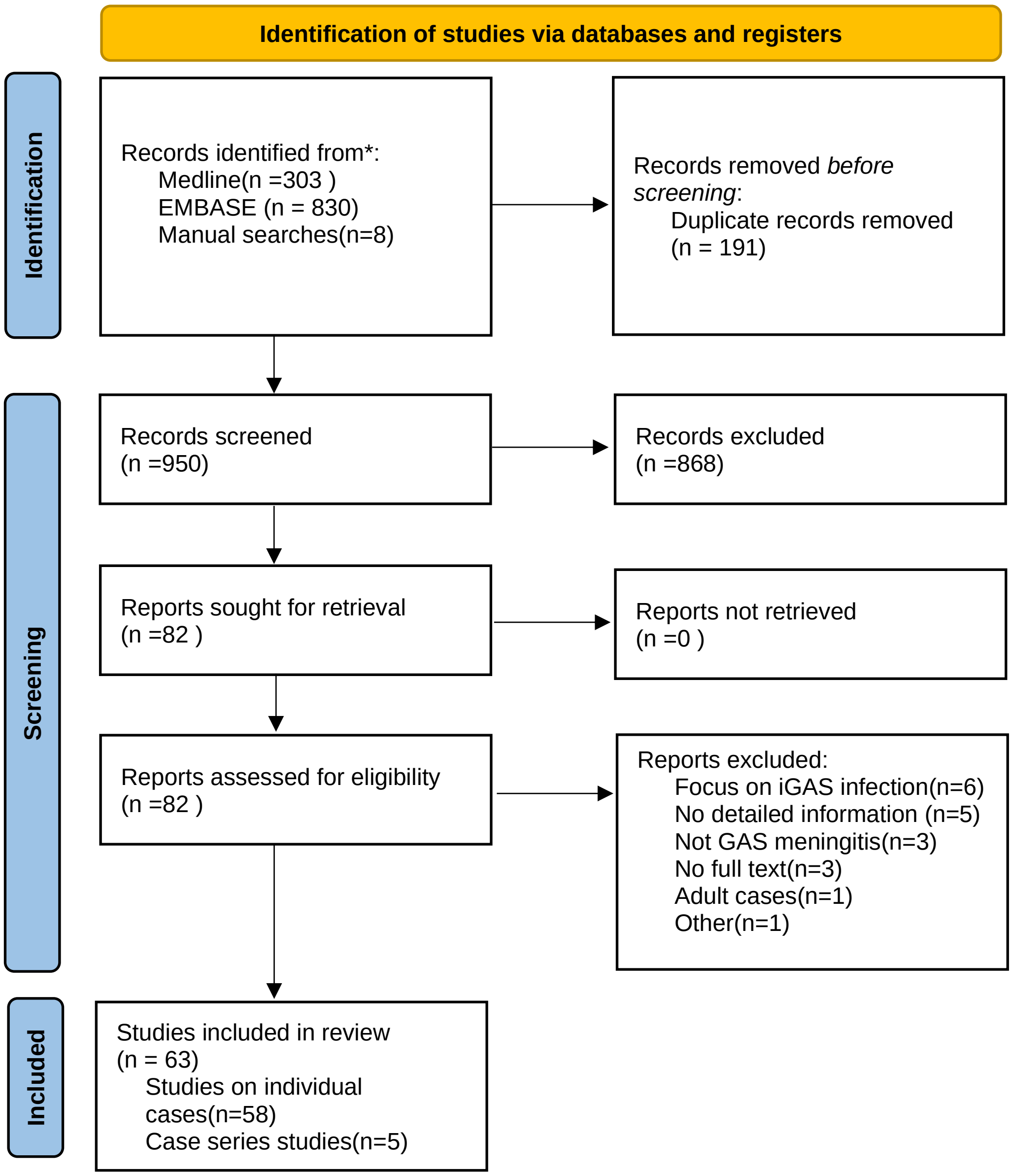

Supplement: Supplementary file 2 — Supplementary Material 2 [file 10096_2024_4863_MOESM2_ESM.pdf]
