# Supplementary material for: Group A Streptococcal meningitis in children: a short case series and systematic review
Source: Eur J Clin Microbiol Infect Dis. 2024 Jun 6;43(8):1517–31. doi: 10.1007/s10096-024-04863-2 (PMC11271352; doi:10.1007/s10096-024-04863-2)
Supplement: Supplementary file 3 — Supplementary Material 3 [file 10096_2024_4863_MOESM3_ESM.pdf]

The European Journal of Clinical Microbiology & Infectious Diseases

Group A Streptococcal Meningitis in Children: A Short Case Series and Systematic Review

Zhen-zhen Dou MD, Wanrong Li MMed, Hui-Li Hu, MBBS, Xin Guo, MMed, Bing Hu, MMed, Tian-ming Chen, MMed, He-ying Chen, MBBS, Ling-yun Guo, MD, Gang Liu, MD

Supplemental table Detailed information of sequela of survivors

Published time	Author	Sequela	Duration of follow-up
1976	Burech,D.L	Persistent unilateral fourth nerve palsy	No discription
1981	Brown, C	Learning difficulty, abnormality of spatial orientation and deficit in short-term memory	2 years
1983	Murphy, D. J.	Hemianopsia	19 days
1983	Murphy, D. J.	Functioning at a 3- month-old level with psychomotor retardation, seizures, microcephaly and optic atrophy	2 years
1984	Rotimi V O,	Poor vision, impaired hearing and severe mental retardation	No description
1988	Harnden, A.	Profound sensorineural hearing loss	2 years
1992	Levy, E. N	Minimal impairment of fine motor skills and mild partial nominal aphasia	6 weeks
1998	Moses, A. E.;	Decreased abdominal amuscle tone, mild adductor spasm and relative weakness of left arm	18 months
2001	Steppberger K,	Oculomotor nerve palsy	No description
2004	Rezvani, M.	Intractable seizures, profoundly delayed, only able to roll	12 months
2005	Ulloa-Gutierrez, R.	Mild fine-motor developmental delay	No description
2010	Bruun T	Headache, possible attention disorder	1 month
2011	Hayashi, A	Hemianopsia, seizures	1 year
2012	Paul, S. P.	Sensorineural hearing loss	6 months
2021	Lee J	Profound sensorineural hearing loss	2 weeks
2023	Nack, T.	Epilepsy and developmental delay	No description
This study		paralysis, mental retardation and mood disorders, sensorineural hearing loss	2 years
This study		had difficulty in study, epilepsy (controlled with drugs)	3 years
