# Supplementary material for: Group A Streptococcal meningitis in children: a short case series and systematic review
Source: Eur J Clin Microbiol Infect Dis. 2024 Jun 6;43(8):1517–31. doi: 10.1007/s10096-024-04863-2 (PMC11271352; doi:10.1007/s10096-024-04863-2)
Supplement: Supplementary file 4 — Supplementary Material 4 [file 10096_2024_4863_MOESM4_ESM.pdf]

The European Journal of Clinical Microbiology & Infectious Diseases

Group A Streptococcal Meningitis in Children: A Short Case Series and Systematic Review

Zhen-zhen Dou MD, Wanrong Li MMed, Hui-Li Hu, MBBS, Xin Guo, MMed, Bing Hu, MMed, Tian-ming Chen, MMed, He-ying Chen, MBBS, Ling-yun Guo, MD, Gang Liu, MD

Last run via:	OVID
Search Screen:	Advanced Search
Databases:	Medline
Date of last search:	24th Feb 2024
Filters	Publication date from 1000/1/1 to 2024/2/24
Considering that some case reports included both paediatric cases and adult cases, we did not set age limitation in search strategy	

Search number	Query	Results
1	Streptococcus pyogenes/	19346
2	group A strep* or strep* group A or strep* pyogenes	19587
3	meningitis	138126
4	(Group A Streptococcal meningitis[MeSH Terms]) OR (Group A Streptococcal meningitis[Title/Abstract])	51
5	(Streptococcus pyogenes meningitis[MeSH Terms]) OR (Streptococcus pyogenes meningitis[Title/Abstract])	166
6	1 OR 2	19587
7	6 AND 3	294
8	4 OR 5 OR 7	303

Last run via:	OVID
Search Screen:	Advanced Search
Databases:	EMBASE
Date of last search:	24th Feb 2024
Filters	Publication date from 1000/1/1 to 2024/2/24
Considering that some case reports included both paediatric cases and adult cases, we did not set age limitation in search strategy	

Search number	Query	Results
1	'streptococcus pyogenes'	22359
2	'group a strep*'	11531
3	'strep* group a'	8776
4	'strep* pyogenes'	22425
5	'group a streptococcal meningitis':ti,ab,kw	57
6	'group a streptococcal meningitis'/exp	16
7	'streptococcus pyogenes meningitis':ti,ab,kw	18
8	'streptococcus pyogenes meningitis'/exp	20
9	'meningitis'	115605
10	1 OR 2 OR 3 OR 4	33077
11	10 AND 9	830
12	5 OR 6 OR 7 OR 8	82
13	11 OR 12	830
