# Supplementary material for: Group A Streptococcal meningitis in children: a short case series and systematic review
Source: Eur J Clin Microbiol Infect Dis. 2024 Jun 6;43(8):1517–31. doi: 10.1007/s10096-024-04863-2 (PMC11271352; doi:10.1007/s10096-024-04863-2)
Supplement: Supplementary file 5 — Supplementary Material 5 [file 10096_2024_4863_MOESM5_ESM.pdf]

The European Journal of Clinical Microbiology & Infectious Diseases

Group A Streptococcal Meningitis in Children: A Short Case Series and Systematic Review

Zhen-zhen Dou MD, Wanrong Li MMed, Hui-Li Hu, MBBS, Xin Guo, MMed, Bing Hu, MMed, Tian-ming Chen, MMed, He-ying Chen, MBBS, Ling-yun Guo, MD, Gang Liu, MD

Detailed statistics outcomes

Items	Survivors	deaths	P value
Aged 0-2 years	25	10	0.016
Male	30	3	0.303
Neurosurgery	23	3	0.820
Systemic complication[a]	9	5	0.011
Intracranial complications	30	3	0.773
Shock	4	6	<0.001
Positive blood culture	20	3	0.876
Hematogenic cases	35	11	0.055
