# Supplementary material for: Group A Streptococcal meningitis in children: a short case series and systematic review
Source: Eur J Clin Microbiol Infect Dis. 2024 Jun 6;43(8):1517–31. doi: 10.1007/s10096-024-04863-2 (PMC11271352; doi:10.1007/s10096-024-04863-2)
Supplement: Supplementary file 6 — Supplementary Material 6 [file 10096_2024_4863_MOESM6_ESM.pdf]

The European Journal of Clinical Microbiology & Infectious Diseases

Group A Streptococcal Meningitis in Children: A Short Case Series and Systematic Review

Zhen-zhen Dou MD, Wanrong Li MMed, Hui-Li Hu, MBBS, Xin Guo, MMed, Bing Hu, MMed, Tian-ming Chen, MMed, He-ying Chen, MBBS, Ling-yun Guo, MD, Gang Liu, MD

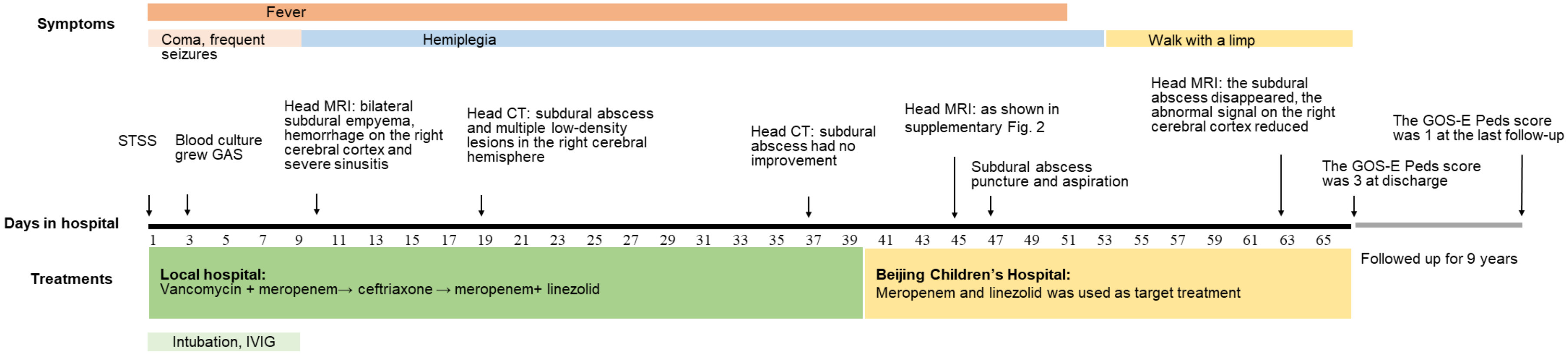

Supplementary Fig. 1 The clinical course of case 1

STSS: Streptococcal Toxic shock syndrome; GAS: group A streptococcus; IVIG: intravenous immunogloblin; GOS-E Peds Score: Glasgow Outcome Scale–Extended Paediatric Reversion

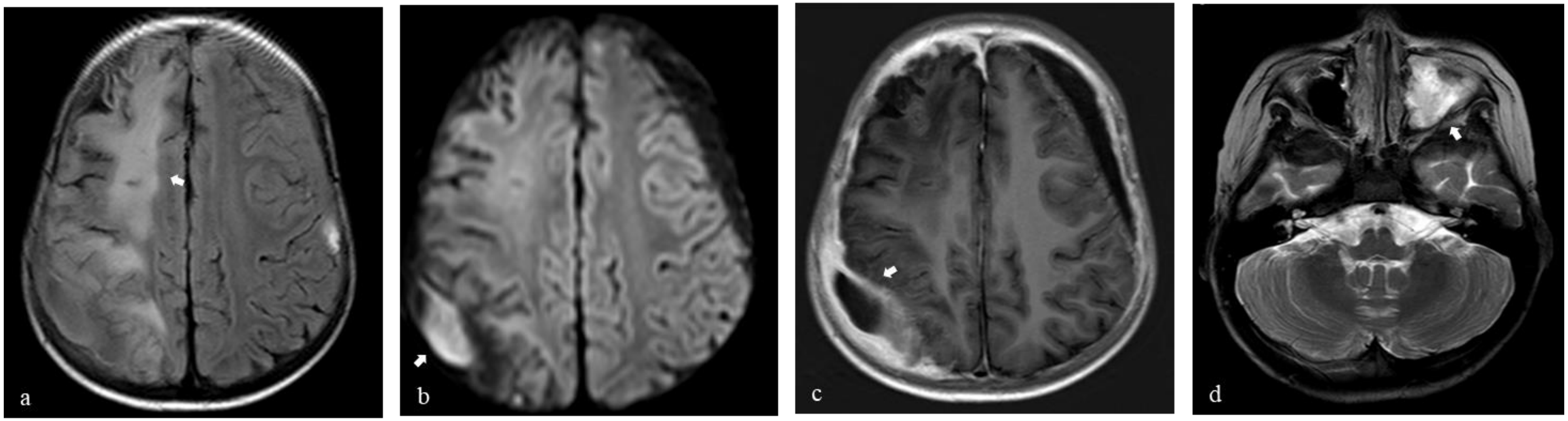

Supplementary Fig. 2 The head MRI of case 1 on day 45

a(Flair images): abnormal signals in right frontal parietal lobe(*arrow*). b(Diffusion Weighted Images) : subdural abscess near the right parietal lobe(*arrow*). c(T1-weighted contrast enhanced images): meningeal enhancement(*arrow*). d(T2-weighted images): opacification in left maxillary sinus(*arrow*).

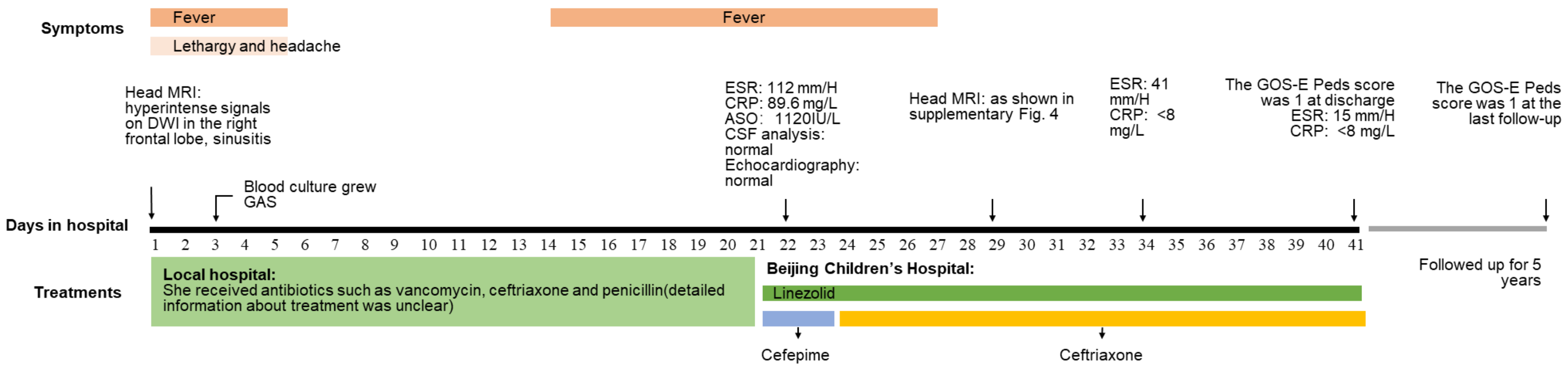

Supplementary Fig. 3 The clinical course of case 2

DWI: diffusion weighted imaging; GAS: *Group A streptococcus;* ESR: Erythrocyte sedimentation rate; CRP: C-reactive protein; ASO: anti streptolysin; CSF: cerebrospinal fluid; GOS-E Peds Score: Glasgow Outcome Scale–Extended Paediatric Reversion.

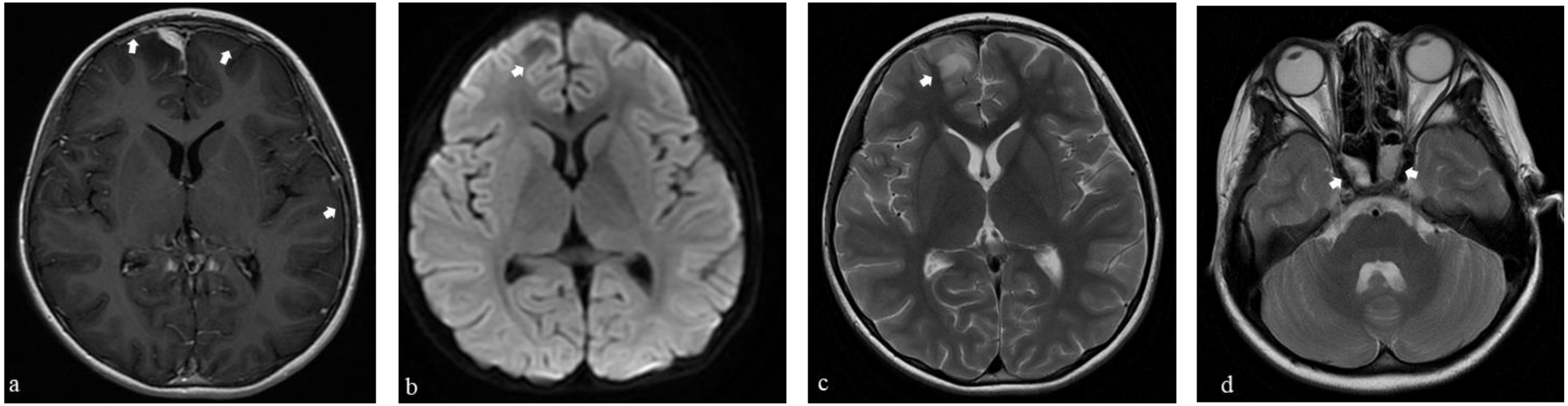

Supplementary Fig. 4 The head MRI of case 2 on day 29

a(T1-weighted contrast enhanced images): meningeal enhancement(*arrows*); b(Diffusion Weighted Images) and c(T2-weighted images): patchy abnormal signals in the right frontal lobe(*arrows*); d(T2-weighted images): sphenoiditis(*arrows*).

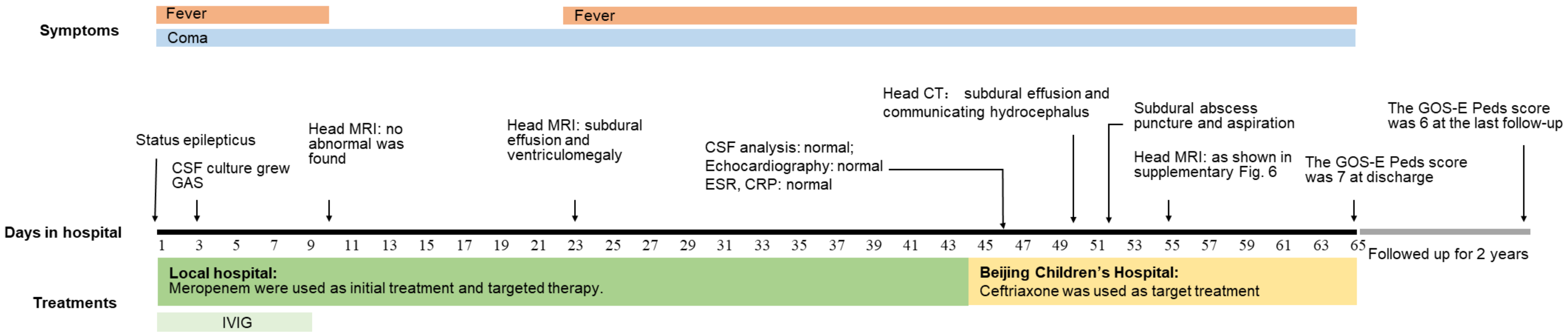

Supplementary Fig. 5 The clinical course of case 3

CSF: cerebrospinal fluid; GAS: *Group A streptococcus;* ESR: Erythrocyte sedimentation rate; CRP: C-reactive protein; GOS-E Peds Score: Glasgow Outcome Scale–Extended Paediatric Reversion.

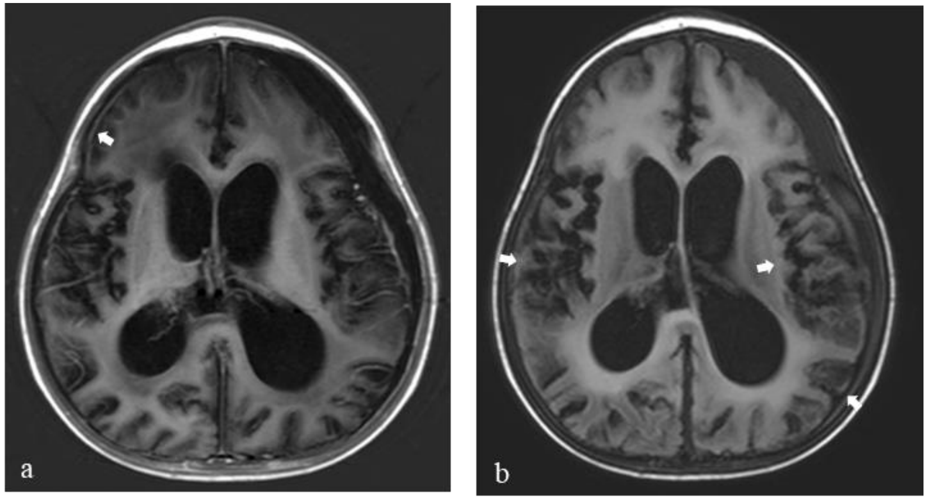

Supplementary Fig. 6 The head MRI of case 3 on day 55

A(T1-weighted contrast enhanced images), B(Flair images): extensive cortical necrosis, subdural effusion and communicating hydrocephalus(*arrows*).

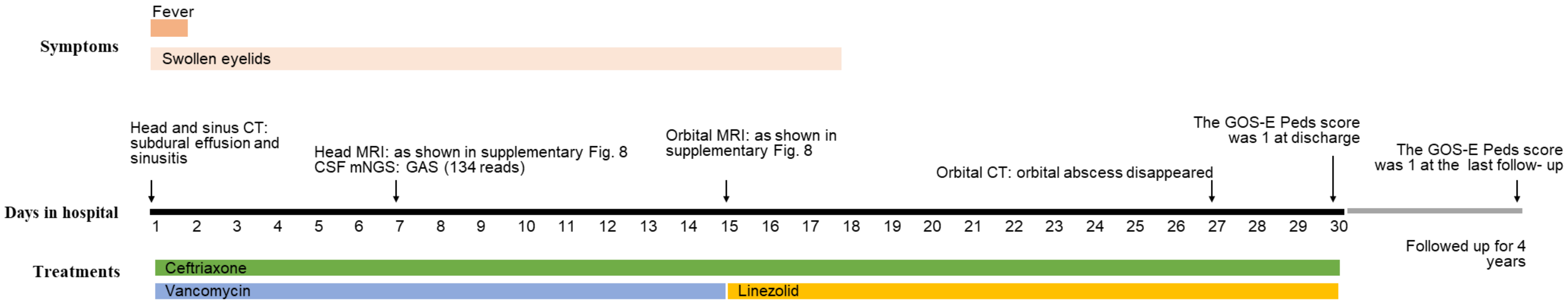

Supplementary Fig. 7 The clinical course of case 4

GAS: *Group A streptococcus;* mNGS: metagenomic next generation sequencing; GOS-E Peds Score: Glasgow Outcome Scale–Extended Paediatric Reversion.

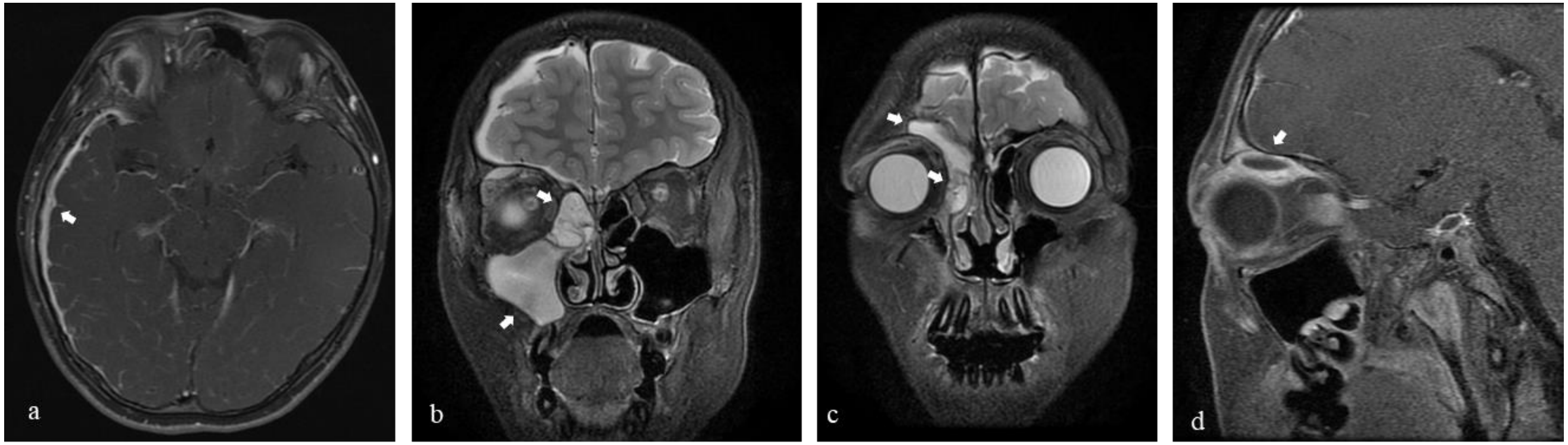

Supplementary Fig. 8 The head MRI of case 4 on day 7 and the orbital MRI of case 4 on day 15
a(T1-weighted contrast enhanced images): the head MRI on day 7, showed enhanced leptomeninges is presented in right hemisphere(*arrow*).
b-c(T2-weighted images) and d(Flair images): the orbital MRI on day 15, showed opacification is present in the right sphenoidal, maxillary, ethmoid, and frontal sinuses, along with orbital cellulitis and an orbital abscess(*arrows*).

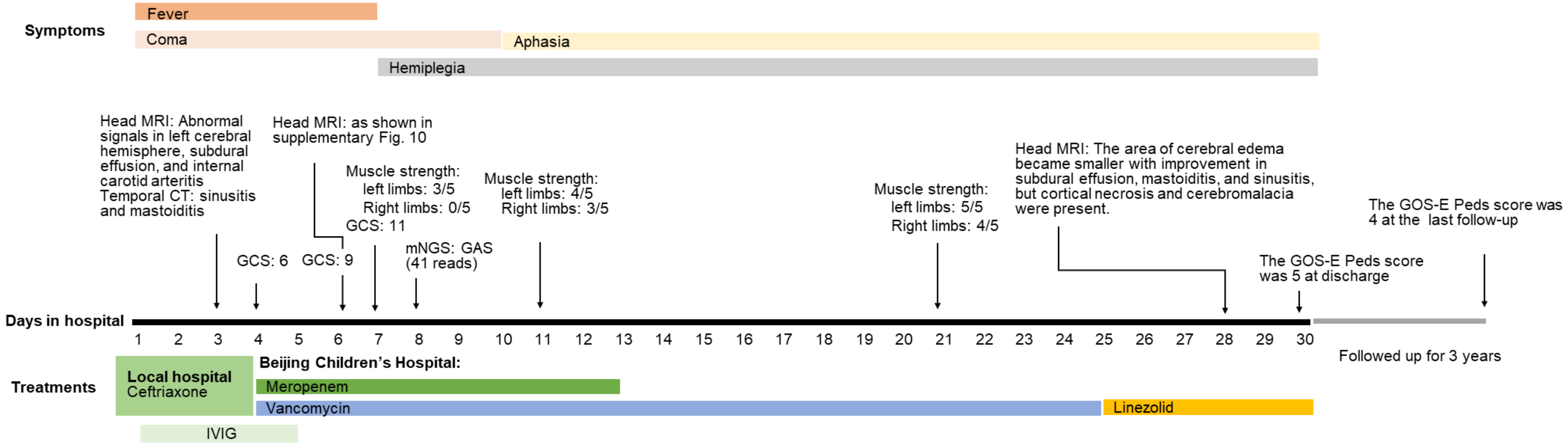

Supplementary Fig. 9 The clinical course of case 5

GCS: Glasgow Coma Score; mNGS: metagenomic next generation sequencing; GAS: *Group A streptococcus;* GOS-E Peds Score: Glasgow Outcome Scale–Extended Paediatric Reversion.

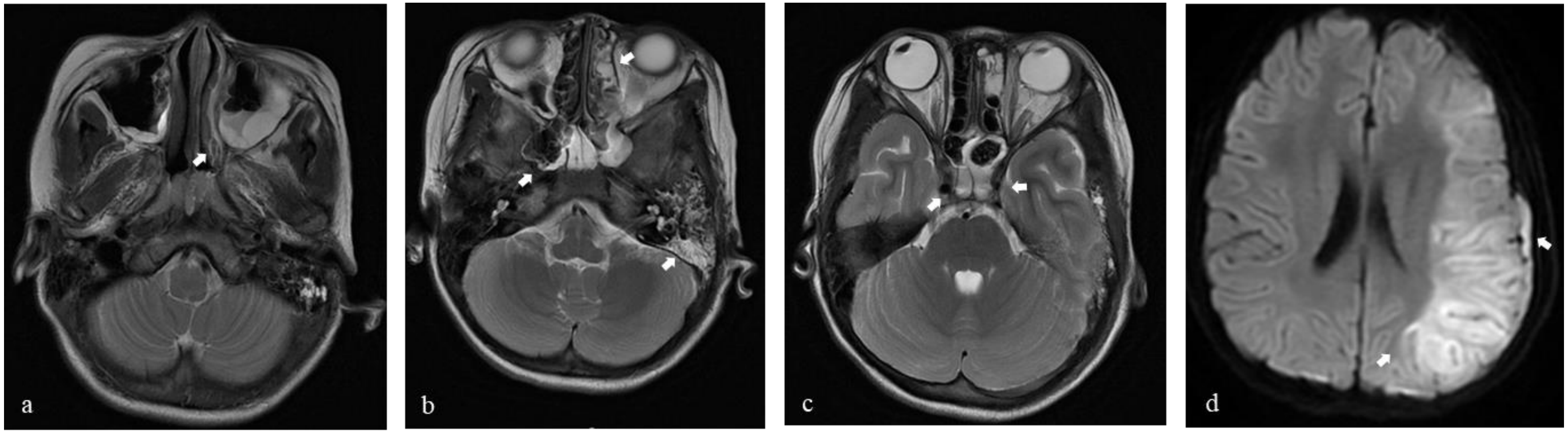

Supplementary Fig. 10 The head MRI of case 5 on day 6

a, b(T2-weighted images): sinusitis and mastoiditis; c (T2-weighted images): internal carotid arteritis, d(Diffusion Weighted Images): cerebral infarction on the left cerebral hemisphere and subdural empyema.
